# Autogenic Training for Reducing Chronic Pain: a Systematic Review and Meta-analysis of Randomized Controlled Trials

**DOI:** 10.1007/s12529-021-10038-6

**Published:** 2021-10-27

**Authors:** Antonia Kohlert, Katharina Wick, Jenny Rosendahl

**Affiliations:** 1 Institute of Psychosocial Medicine, Psychotherapy and Psychooncology, Jena University Hospital, Stoystr. 3, 07743 Jena, Germany; 2 University of Applied Health Sciences, Gera, Germany

**Keywords:** Autogenic training, Chronic pain, Systematic review, Meta-analysis, Randomized controlled trials

## Abstract

**Background:**

Autogenic training (AT) is frequently used as therapeutic approach in multimodal pain therapy. The aim of this systematic review and meta-analysis is to investigate the efficacy of AT in individuals suffering from chronic pain in comparison to passive and active control groups.

**Methods:**

A comprehensive literature search in Medline, Web of Science, PsycInfo, and PubPsych and manual searches (last search April 7, 2021) were conducted to locate randomized controlled trials (RCTs). Treatment guidelines and references of relevant articles and previous reviews were checked. ProQuest Dissertations and Theses Full Text database, DART-Europe E-theses Portal, Networked Digital Library of Theses and Dissertations (NDLTD), and the Theses Database of the German National Library were screened to identify any unpublished material.

**Results:**

A total of 13 eligible studies (*k* = 15 comparisons) including 576 participants were identified. Random-effects meta-analyses revealed a significantly positive, moderate effect of AT on the primary outcome pain compared to passive control groups (*g* = 0.58, 95% *CI* [0.36; 0.79], *k* = 9, *I*^*2*^ = 0%). In comparison with other psychological interventions, no difference was found (*g* = − 0.05, 95% CI [− 0.30; 0.20], *k* = 6, *I*^*2*^ = 0%). Sensitivity analyses proved the robustness of findings. Overall risk-of-bias judgment was ‘some concerns’ in the majority of studies.

**Conclusions:**

Beneficial effects of AT on pain reduction were demonstrated, but findings are prone to bias. Furthermore, high methodological quality RCTs are needed to strengthen the promising evidence of AT for individuals with chronic pain.

**Supplementary Information:**

The online version contains supplementary material available at 10.1007/s12529-021-10038-6.

## Introduction

According to International Association for the Study of Pain, pain is defined as an unpleasant sensory and emotional experience associated with, or resembling that associated with, actual or potential tissue damage [1]. Pain is generally classified into two categories. Acute pain serves as a warning signal and can be defined as a reaction to tissue damage triggered by aversive external stimuli or endogenous processes [2, 3]. As far as adequate treatment of the cause is possible, acute pain is reversible [4]. In contrast, chronic pain either persists after the injury is healed or it is associated with a chronic illness [5]. In pain research, chronic pain includes both persistent and recurring pain with at least 3- to 6-month duration [6].

Pain is a significant physical strain for the individual, which is associated with decreased quality of life, reduced job productivity, and increased absence from work [7]. The direct and indirect health care costs of chronic pain disorders in European member states are estimated at 2–3% of gross domestic product across the EU [8, 9]. For 2016, this was approximately 441 billion euros [10]. Chronic pain carries a significant burden for employees, employers, and society, and the adverse consequences of chronic pain with its substantial negative impact on work-related outcomes are often underestimated [11]. According to a large-scale internet-based survey on prevalence and attributes of pain experiences in the UK, France, Spain, Germany, and Italy, one in five respondents had experienced pain in the past few months [7]. For various pain types, high prevalence rates are reported for 19 European countries. At a pan‐European level, back/neck pain was the most prevalent with 40% of survey participants experiencing pain [12].

Particularly in the current Covid-19 pandemic, delaying or discontinuing treatment for individuals suffering from severe chronic pain has negative consequences for them, such as an increase in pain [13]. With the limited availability of multimodal therapeutic approaches in the pandemic, physicians must prescribe pain medications until adequate treatment is available [14]. In summary, because of their high prevalence, relating costs and worsened chronic pain symptoms due to COVID-19 chronic pain has not only high personal, but also clinical and economic relevance for patients, practitioners, and payers.

Oral analgesics are one of the primary treatments for different types of pain because they can be a quick, cheap, and effective solution to the problem of pain [15]. However, the use of pharmaceuticals for pain reduction can lead to various unwanted side effects. In addition to physical side effects such as kidney, liver, or cardiovascular problems, these also include drug dependency as well as sedation and tolerance effects [16]. Non-pharmacological psychological interventions aim at modifying factors that are important in the genesis and maintenance of pain [17]. Meanwhile, these interventions are essential in multimodal pain therapy. In Germany, almost every facility that offers multimodal pain treatment applies at least one relaxation procedure, such as progressive muscle relaxation, biofeedback, and autogenic training (AT), as routine part of pain treatment [18].

In the following study, we focus on AT only, because in comparison to progressive muscle relaxation, the patient is not forced to build up additional muscle tension of the painful muscle sections but can achieve an improvement by directing his/her attention to certain relaxation reactions of the body (for more details, see below). Moreover, AT can be performed without medical supervision. In cases where the patient’s health could be affected using the relaxation exercise such as PMR for pain, physical illness, disabilities, or injury, medical supervision is recommended [19]. Compared to biofeedback, AT is superior because it can be used in everyday life in any situation, whereas with biofeedback, there is a dependence on experts and equipment [19].

AT is a self-relaxation procedure applying passive concentration on certain combinations of psychophysiologically adapted stimuli, developed by Schultz almost 100 years ago [20]. Within AT, participants are trained in auto-suggestive techniques to influence their physical condition [21–23]. In its classic form, AT uses six standard exercises that are trained in individual or group settings over a period of 6 to 8 weeks [24, 25]. Participants sit or lie in a quiet, undisturbed setting and focus on different areas of the body, which are addressed using six suggestive formulas aiming at increasing relaxation and balance between sympathetic and parasympathetic control [26]. Relaxation is suggested to affect pain by reducing tissue oxygen requirement and degrading lactic acid, by relieving skeletal muscle tension and anxiety, and by releasing endorphins [27, 28].

In the past decades, several systematic reviews summarized the evidence on AT for various clinical indications including pain. Within the extensive review of Grawe et al. [29] including more than a thousand psychotherapy studies published up to 1983/84, only 14 trials were controlled AT studies. Based on their results, the authors concluded that the effectiveness of AT has not yet been sufficiently validated compared to other relaxation techniques. At the same time, the first meta-analysis of AT was released, including 24 controlled studies published from 1952 to 1993 [26]. However, pain was not explicitly considered primary outcome, but was included in the aggregate of ‘behavioral and psychological outcomes.’ AT was associated with medium-sized pre-post effect sizes in migraine and tension headache, but this estimation was based on two or five studies, respectively. Stetter and Kupper [25] updated this review in 2002 examining 60 clinical studies published between 1952 and 1999, including 35 randomized controlled trials. Outcomes were grouped as either ‘physiological’ or ‘behavioral and psychological’. Eleven randomized controlled trials were included examining the effects of AT in individuals with tension headache/migraine, providing a significantly positive, medium effect size of *d* = 0.59 (four studies, 251 participants) on all reported outcomes when AT was compared with passive control groups. In comparison to other psychological interventions, there were significantly negative effects of *d* = − 0.25 showing that other psychological treatments performed better than AT in reducing headache/migraine pain (seven studies, 871 participants). Kanji et al. [30] published a systematic review on the effectiveness of AT in individuals with tension headache including five randomized controlled trials and two non-randomized controlled trials. AT was comparable to other types of interventions, and only some studies revealed inferior effects in contrast to biofeedback. The authors concluded that AT is an effective relaxation technique for individuals with pain; however, this was based on a narrative summary only.

Altogether, the efficacy of AT in individuals suffering from pain has been investigated in numerous randomized controlled trials. However, a previous meta-analysis is about 20 years old and has so far only summarized the existing evidence without specifically considering pain as outcome [25]. Hence, the aim of this meta-analysis is to investigate the efficacy of AT in individuals with chronic pain on the primary outcome pain in comparison to waiting list control groups, attention control groups, or control groups that received other psychological interventions. In addition to pain as primary outcome, mental distress and health-related functioning are considered secondary outcomes.

## Methods

### Protocol and Registration

The review was registered at PROSPERO International Prospective Register of Systematic Reviews (CRD42020141812).

### Eligibility Criteria

Randomized controlled trials published in English or German language without restrictions of publication date were included. Eligible studies involved individuals with chronic pain and evaluated the efficacy of AT. AT had to be applied for therapeutic purposes, had to be the only or at least the primary therapeutic method, and could be performed individually or in a group. ‘No treatment’, ‘attention control’, or ‘another treatment’ was considered eligible control groups. Attention control groups were defined as delivering a comparable amount of time and attention without specific therapeutic components. Another treatment included standard care or another type of intervention referred to as relaxation intervention. Primary outcome was pain including measures of, e.g., pain intensity, frequency, and duration. Mental distress (including measures of, e.g., anxiety, depression, well-being, relaxation, comfort) and health-related functioning were considered secondary outcomes. Outcomes reflecting quality of life (pre-specified as secondary outcomes in the review protocol) were classified as mental distress or health-related functioning, depending on the subscales of the quality of life measures. Deviating from the review protocol, we excluded studies on somatoform and acute pain and limited study inclusion to a more homogeneous population of individuals with chronic pain.

### Information Sources and Search

A systematic literature search was performed using the following electronic databases: Medline, Web of Science, PsycInfo, and PubPsych (date last searched: April 7, 2021; see Supplementary material for details of the search strategy). The search strategy specified terms referring to the patient population (e.g., pain*), treatment (e.g., autogenic training, autogenic*, autosuggest*), and study design (e.g., random*, control*). The search strategy was developed with consideration of validated search strategies for retrieving randomized controlled trials [31]. Additionally, relevant treatment guidelines and references of recent reviews, meta-analyses, and primary studies were checked to identify further studies. In order to detect unpublished studies, the ProQuest Dissertations and Theses Full Text database, DART-Europe E-theses Portal, Networked Digital Library of Theses and Dissertations (NDLTD), and the Theses Database of the German National Library were searched.

### Study Selection

Title and abstract of studies identified in the literature search were first screened for eligibility by the first author. In a second step, full texts of the preselected studies were examined in detail for eligibility by two independent researchers (AK, JR). Disagreements were resolved by consensus.

### Data Extraction

The following data were extracted from the included studies: information on publication (e.g., authors, publication year, country of origin), sample characteristics (e.g., sample size, gender, age, type of pain), characteristics of the intervention group (e.g., treatment format, treatment modality, number of sessions, length of sessions, total duration), characteristics of the control group (e.g., type of control group), information on outcomes (e.g., type of outcome category, measure, time point), and statistical data. Descriptive information was coded by the first author. Two authors (AK, JR) extracted information on outcomes and statistics needed for effect size estimation with disagreements resolved by consensus.

### Risk of Bias in Individual Studies

To evaluate various indicators of bias, the current version of the Cochrane Risk of Bias Tool for Randomized Trials (ROB2—revised version from August 2019) was used [32]. Risk of bias was assessed in five distinct domains. Within each domain, one or more signaling questions were answered. Based on defined algorithms, judgments of ‘low risk of bias,’ ‘some concerns,’ or ‘high risk of bias’ were proposed for each domain. The judgments within each domain finally resulted in an overall risk-of-bias judgment per study. Risk of bias was assessed for each study and domain independently by two authors (AK, KW). Disagreement in judgments was resolved either via discussion or another author (JR) was called to adjudicate the final judgment.

### Summary Measures

Between-group effect sizes (Hedges’ *g*) were computed for each comparison, assessment time point, and outcome of interest. Hedges’ *g* represents the standardized mean difference calculated by subtracting the posttreatment mean of the intervention group from the posttreatment mean of the control group, dividing the result by the pooled standard deviation, and multiplied by a small-sample bias correction factor [33]. If means and standard deviations were not reported, other statistics (*F*, *t*, or *p* value) were used to calculate effect sizes. For dichotomous outcomes, log odds ratios were calculated and converted to Hedges’ *g* in order to pool across different effect size formats [34]. The magnitude of Hedges’ g was interpreted within the same framework as Cohen’s *d*, regarding 0.20, 0.50, and 0.80 as small, medium, and large effects between two contrasted groups, respectively [35]. Positive effect sizes indicate that AT was superior to the comparison treatment, whereas negative effect sizes suggest superiority of the comparison treatment. All summary measures are reported with a 95% confidence interval (CI). The software Comprehensive Meta-Analysis (CMA, Biostat. Inc. Version 3) was used for computing effect sizes and performing data analyses.

### Data Synthesis

Outcome data were meta-analyzed using a random-effects approach. The generic inverse variance method with heterogeneity estimated using the DerSimonian-Laird method was applied [36]. In case of multiple comparisons within one study (two control groups were compared against one shared intervention group), each pairwise comparison was included separately in the meta-analysis as proposed by Higgins et al. [37]: for dichotomous outcomes, both the number of events and the total number of patients in the shared intervention group were divided evenly among the pairwise comparisons. For continuous outcomes, only the total number of patients was divided and statistical parameters were left unchanged. If multiple outcomes were reported within one outcome domain (e.g., two measures of pain), effect sizes were aggregated within domains for each unit of analysis and correlations between outcomes were set at 0.50 [38]. All pooled effect sizes Hedges’ *g* were additionally transformed into numbers needed to treat (*NNT*) [39], representing the number of patients one would need to treat with the intervention in order to have one more patient to have an outcome better than a randomly selected one in the control group (note that negative *NNT* values refer to the number needed to harm).

Statistical heterogeneity across studies was assessed with χ^2^ heterogeneity tests (Cochrane’s *Q*) and *I*^*2*^ statistic [40]. *I*^*2*^ describes the percentage of the variability in effect estimates that is due to heterogeneity rather than chance, with values from 0 to 40% indicating no important heterogeneity, 30 to 60% moderate, 50 to 90% substantial, and 75 to 100% considerable heterogeneity, respectively [41]. Additionally, 95% prediction intervals were computed representing the potential effect in a future study that is similar to the studies in the meta-analysis [41, 42].

### Risk of Bias Across Studies

In order to address potential publication bias, funnel plots were visually inspected and Egger’s regression test for funnel plot asymmetry was performed [43]. Duval and Tweedie’s trim and fill procedure was used to determine whether small studies with non-significant effects were underrepresented in the meta-analysis [44]. Possible missing studies were imputed and the effect size estimate was recalculated. Additionally, Classic fail-safe *N* [45] was computed estimating the number of studies with a null effect that would be needed to increase the *p* value for the meta-analysis to above 0.05.

### Additional Analysis

Subgroup analyses and meta-regression analyses were planned for various pre-specified variables, given a sufficient number of available studies (per group). To test the robustness of effect size estimates, sensitivity analyses were performed by excluding studies with children, approximated effect sizes, and studies with high risk of bias in any domain.

## Results

### Study Selection

**Fig. 1:**
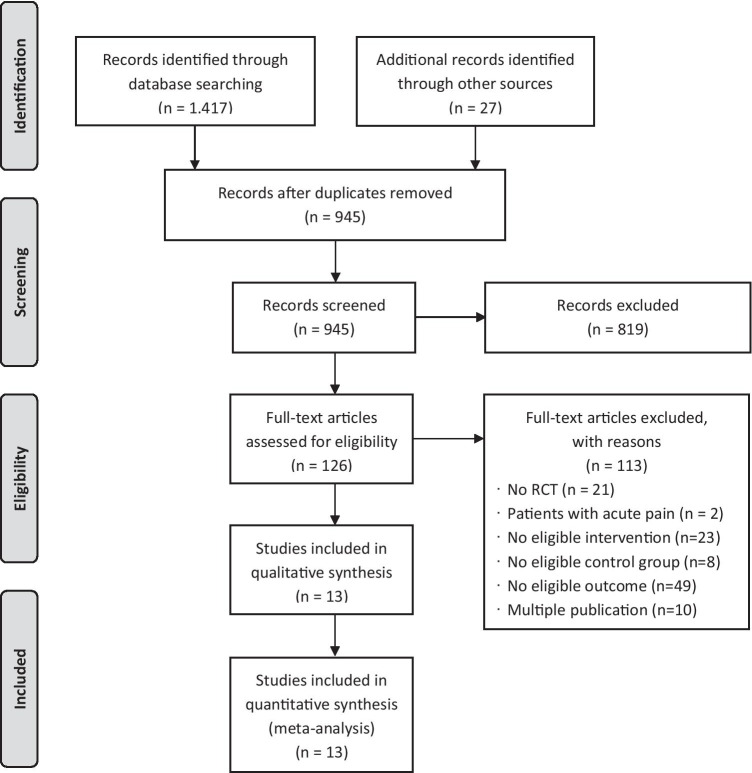
Flow chart of the study selection process

A total of 945 records were screened and finally *N* = 13 RCTs were included in the meta-analysis. Figure 1 contains the flow chart of the study selection process.

### Study Characteristics

All included studies were published in English between 1986 and 2012 (Table 1). Among the primary studies, three each were from the Netherlands and the USA, two from France, and one each from Germany, Australia, Japan, Italy, and the UK. The 13 included RCTs provided *k* = 15 comparisons between an AT intervention and a control group, incorporating a total of 576 randomized participants, with 256 allocated to AT and 320 to control groups, respectively. Mean age of the participants ranged from 12 to 71 years (*M* = 38.1, *SD* = 15.3), and 60% were female. All studies included participants with chronic pain. Of the included studies, eight examined individuals with headache (tension headache, migraine), and one trial each investigated chest pain in individuals with cardiac syndrome, multiple sclerosis, cancer, rheumatoid arthritis, and irritable bowel syndrome, respectively.

**Table 1:** Characteristics of the included studies

Study	Country	Type of pain	Mean age (years)	% female	Intervention group				Control group		Outcome
					AT format	Dose (min)	Modality	*n*	Type of control group	*n*	
Asbury et al. (2009) [46]	UK	Chest pain in patients with cardiac syndrome X	57.1	100	Group	720	Live	26	TAU	27	Pain, MD, HRF
Bernateck et al. (2008) [47]	Germany	Rheumatic pain	51.6	68	Group	180	Live + audio recording	22	Active intervention (auricular electroacupuncture)	22	Pain, HRF
Collet et al. (1986) [48]	France	Tension headache	39.7	52	Group	250	Audio recording	15	Active intervention (galvanic skin response biofeedback)	16	Pain, MD
Engel et al. (1990) [49]	USA	Mixed-headache disorders	12.9	62	Individual	300	Live + audio recording	5	TAU	5	Pain
									Active Intervention (PMR)	5	Pain
Janssen et al. (1986) [50]	Netherlands	Migraine and tension headache	33.4	63	Group	720	Live	5	Active Intervention (PMR)	5	Pain
Labbé (1995) [51]	USA	Migraine	12	28	n.r	450	Live	10	TAU	10	Pain
Mantovani et al. (1996) [52]	Italy	Pain in cancer patients	70.7	42	Group	1560	Live	24	AttCG	23	Pain, MD, HRF
Pickering et al. (2012) [53]	France	Chronic headache	39	60	Individual	480	Live + audio recording	29	TAU	29	Pain
Sargent et al. (1986) [54]	USA	Migraine	35.7	59	Group	120	Live	24	TAU	24	Pain, HRF
Shinozaki et al. (2010) [55]	Japan	Pain in patients with irritable bowel syndrome	31.6	52	Individual	280	Live + audio recording	11	AttCG	10	Pain, MD, HRF
Spinhoven et al. (1992) [56]	Netherlands	Tension headache	36	61	Individual	180	Live + audio recording	23	Active Intervention (Self-hypnosis)	23	Pain, MD
Sutherland et al. (2005) [57]	Australia	Pain in patients with multiple sclerosis	42.2	77	Group	n.r	Live	14	TAU	12	Pain, MD, HRF
ter Kuile et al. (1994) [58]	Netherlands	Headache	33.6	57	Individual	360	Live + audio recording	48	TAU	57	Pain, MD
									Active Intervention (Self-hypnosis)	52	Pain, MD

*AttCG* attention control group; *audio recordings* AT was delivered via recordings on tape or CD; *HRF* health-related functioning; *live* AT was delivered personally by an AT therapist; *MD* mental distress; *min* minutes; *n* randomly allocated participants; *n.r.* not reported; PMR progressive muscle relaxation; *TAU* treatment as usual

AT was either delivered individually (*n* = 5) or in groups (*n* = 7, no information about treatment format in *n* = 1). The number of participants in AT groups ranged from three to 15. In six studies, AT was personally instructed; in one trial, instructions were provided by audio recordings; and another six studies used a combination of personal instruction and audio recordings for home exercises. The number of AT sessions ranged from four to 26 (*Mdn* = 8, *IQR* = 6–10), with a session length of 15 to 90 min (*Mdn* = 60, *IQR* = 35–60). Accordingly, total duration of AT varied from 120 to 1560 min (*Mdn* = 300, *IQR* = 250–720).

AT was contrasted against passive control groups in *k* = 9 comparisons (*k* = 6 treatment as usual, *k* = 3 attention control groups), and against active control groups in *k* = 6 comparisons (*k* = 2 progressive muscle relaxation, *k* = 2 self-hypnosis, *k* = 1 auricular electroacupuncture, *k* = 1 galvanic skin response biofeedback). The primary outcome pain (pain intensity, frequency of pain, absence of pain, pain duration, global pain ratings) was reported in all 15 comparisons. Mental distress (e.g., anxiety, depression, mental distress, quality of life, well-being) was measured in *k* = 8 comparisons, and effects on health-related functioning (e.g., functional status, disability, general health) were provided in *k* = 6 contrasts. In two studies [49, 54], effect sizes had to be estimated based on sample size and *p* value.

### Risk of Bias in Individual Studies

**Fig. 2:**
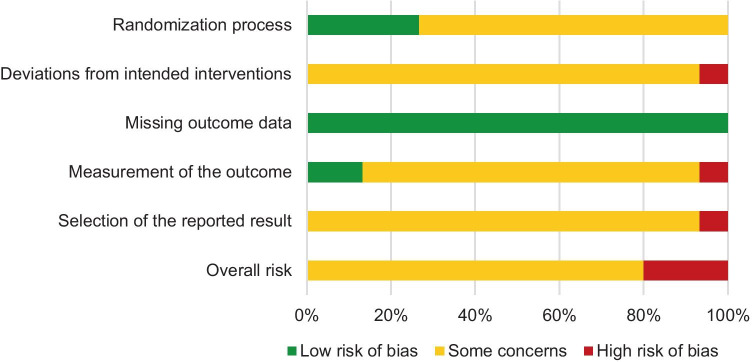
Summary of risk-of-bias judgments of the included studies

A summary of risk-of-bias judgments for the included studies is shown in Fig. 2, and detailed information about single study ratings is provided in Supplementary Table 1. The risk of bias was mainly judged as ‘some concerns’ for the single bias domains, mostly due to missing information in the studies. ‘High risk’ of bias within single domains was rated for three studies [46, 47, 51]. Accordingly, overall risk-of-bias judgment was ‘some concerns’ in ten studies and ‘high risk’ of bias in three trials.

### Results of Individual Studies and Synthesis of Results

#### Primary Outcome

**Fig. 3:**
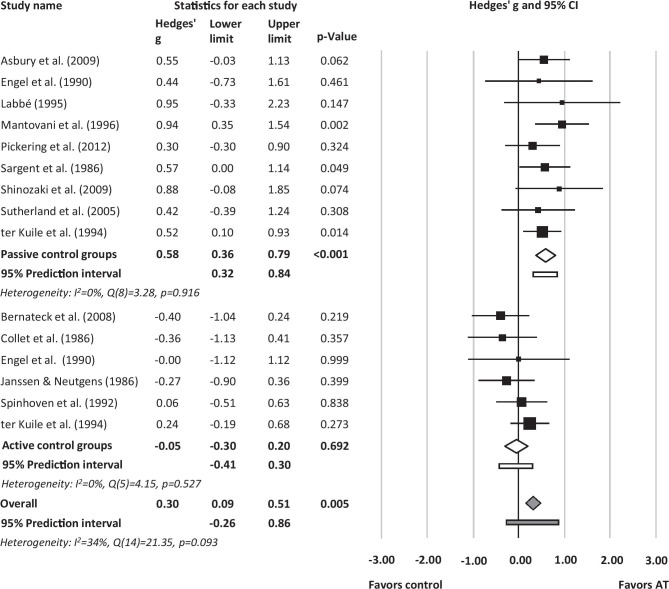
Forest plot of study effects, subgroup effects for passive/active control groups, and total effect of autogenic training for the primary outcome pain

Effect sizes of the included studies for the primary outcome pain measured posttreatment ranged from *g* = − 0.40 [47] to *g* = 0.95 [51]. No statistical outliers (defined as effect sizes with confidence intervals not overlapping with the confidence interval of the pooled effect [59]) emerged. The overall effect was small and significant in favor of AT, *g* = 0.30 (95% *CI* [0.09;0.51], *p* = 0.005, *k* = 15, *NNT* = 5.76). Heterogeneity was moderate, *I*^*2*^ = 34% (*Q* = 21.35, *df* = 14, *p* = 0.093). The 95% prediction interval representing the potential effect in a future study that is similar to the studies in the meta-analysis was − 0.26 to 0.86 (Fig. 3).

#### Passive/Active Control Groups

Efficacy of AT significantly differed as a function of control group type (*p* < 0.001). When contrasted against passive control groups, AT showed larger effects (*g* = 0.58, 95% *CI* [0.36;0.79], *p* < 0.001, *k* = 9, *NNT* = 3.09) than in comparison to active control groups (*g* = -0.05, 95% *CI* [-0.30;0.20], *p* = 0.692, *k* = 6, *NNT* = − 35.23). There was no heterogeneity in both groups (*I*^*2*^ = 0%). The 95% prediction interval for contrast against passive control groups was 0.32 to 0.84, and for active control groups − 0.41 to 0.30, respectively (Fig. 3).

#### Follow-up

Effects for pain remained stable at follow-up with *g* = 0.22 (95% *CI* [− 0.20;0.65], *p* = 0.306, *k* = 6) across all comparisons, *g* = 0.64 (95% *CI* [0.11;1.17], *p* = 0.019, *k* = 3) for contrasts against passive control groups, and *g* = − 0.11 (95% *CI* − 0.55;0.32], *p* = 0.612, *k* = 3) for comparisons against active control groups.

#### Secondary Outcomes

The overall effect for the secondary outcome mental distress at posttreatment was non-significant, *g* = 0.02 (95% *CI* [− 0.18;0.22], *p* = 0.832, *k* = 8) with no heterogeneity (*I*^*2*^ = 0). There was no difference in effects on mental distress when AT was compared to passive or active control groups (*p* = 0.693; Table 2). The effect of AT for the secondary outcome health-related functioning at posttreatment was also found to be non-significant, *g* = 0.05 (95% *CI* [− 0.47;0.57], *p* = 0.855, *k* = 6) with substantial heterogeneity (*I*^*2*^ = 73%). Differences between passive and active control groups were significant (*p* < 0.001) though based only on one comparison against active control groups (Table 2).

**Table 2:** Effects of autogenic training for secondary outcomes

	*k*	*g*	95% *CI*	*p (g)*	*Q*	*p (Q)*	*I*^*2*^
Mental distress	8	0.02	− 0.18; 0.22	0.832	2.13	0.952	0%
Passive control groups	5	− 0.01	− 0.26; 0.24	0.934	0.64	0.959	0%
Active control groups	3	0.07	− 0.24; 0.38	0.660	1.34	0.512	0%
Health-related functioning	6	0.05	− 0.47; 0.57	0.855	18.87	0.002	73%
Passive control groups	5	0.31	0.02; 0.60	0.033	1.31	0.861	0%
Active control groups	1	− 1.30	− 2.00; − 0.60	< 0.001	-	-	-

*k* number of comparisons; *g* Hedges’ g; *95% CI* 95% confidence interval; Q, p(Q), I^2^, test statistics for heterogeneity

### Risk of Bias Across Studies

Visual inspection of the funnel plot did not provide an asymmetry of studies (Supplementary Fig. 1). Trim and fill analysis revealed no missing studies. Furthermore, Egger’s regression test did not indicate a risk for publication bias (*β* = − 0.06; *t*(13) = 0.05; *p* = 0.958). Classic fail-safe *N* analysis shows that the result is fairly robust as 34 new studies would be needed to bring the *p* value above 0.05.

### Additional Analysis

For the primary outcome pain, we found no impact of intervention format (individual vs. group setting), intervention mode (live vs. audio recordings or audio + live), or duration of the AT intervention (Supplementary Table 2). Sensitivity analyses proved the robustness of findings. Sensitivity analyses revealed the stability of results when excluding studies with children, approximated effect sizes, and studies with high risk of bias in any domain (Supplementary Table 3).

## Discussion

### Summary of Evidence

The aim of this meta-analysis was to summarize the existing evidence on the efficacy of AT on chronic pain and to quantify the effects on the primary outcome pain and the secondary outcomes mental distress and health-related functioning in comparison to passive and active control groups. Initially, we aimed to determine the effect of AT on somatoform and acute pain as well, but our search yielded only two studies on acute pain [60, 61] and no study on somatoform pain. Since meta-analytical calculations cannot be interpreted with this small number, we focused on chronic pain. A total of 13 eligible randomized controlled studies including 15 comparisons were identified. Across all studies, small effects of AT on the reduction of pain were found. Compared to passive control groups, the effect of AT on pain was significant and moderate with no heterogeneity across the individual studies. This corresponds to a number needed to treat of 3.1, indicating that three individuals would need to be treated with AT in order to have one patient to have better change in pain than a randomly selected one in the control group. A possible future study comparing AT with a passive control group that is similar to the studies in the meta-analysis is estimated to bring an effect on pain in the range of small to large size. Additionally, no difference on pain between AT and other psychological interventions such as progressive muscle relaxation, biofeedback, or self-hypnosis was found (zero effect). This was based on homogeneous effects in the single studies. AT can therefore be regarded as a suitable method for individuals with chronic pain. Besides its effectiveness, AT has advantages in comparison to PMR and other methods (e.g., no additional muscle tension of the painful muscle sections, no dependence on experts). Additionally, it is well suited to meet the different preferences of individuals and to have a choice to select the method that suits best from a range of different effective methods.

The effects of AT on the primary outcome pain were robust against the exclusion of approximated effect sizes, of studies with children, and of studies with high risk of bias in any domain. Across all studies, we found no indications for a publication bias. No significant effects of AT were found for the secondary outcome variables mental distress and health-related functioning (zero effects).

This is the first meta-analysis on the efficacy of AT specifically on pain outcomes. Our results partially reflect the findings of earlier meta-analyses on the effectiveness of AT on clinical outcome variables. The most recent meta-analysis [25] reported small to medium effect sizes for AT compared to passive control conditions, while in comparison to active control groups, effects were significantly negative (effects in favor of active control treatments). While results of the meta-analysis of Stetter and Kupper [25] were based on eleven studies examining individuals with tension headache/ migraine, we included 13 studies on mixed types of pain with six studies overlapping and seven additionally considered studies published after 2002 or focusing on other types of pain. Our results are also similar to findings of a systematic review by Kanji, White, and Ernst [30] investigating the effects of AT in adults suffering from tension headaches with three overlapping studies. However, results were reported only narratively. Effects of AT found in this review were in the same range as reported in various meta-analyses on the efficacy of other psychological interventions, e.g., hypnosis for individuals with chronic pain [62], acceptance-based interventions for chronic pain [63], psychological interventions (predominantly cognitive-behavioral therapy) for chronic low back pain, arthritis pain, and acute post-operative pain [64–66], for chronic pain in children and adolescents [67] as well as in older adults [68].

Moderator analyses examining the influence of intervention format (group vs. individual), intervention mode (live vs. audio recordings/both), and AT duration did not reveal significant results. However, due to the small number of studies and the associated low statistical power, these results should be interpreted with caution [69].

### Limitations

Although a comprehensive database search, a manual search as well as a search for unpublished studies such as doctoral theses were conducted, it is still possible that eligible trials might have been missed. This could have particularly been the case for studies in which AT had been delivered but not labeled as such by the study authors. The most significant limitation is the small number of included studies—particularly when stratifying the analyses according to contrasts against passive and active control groups—lowering our confidence in the results. Due to an insufficient number of available studies, we had to refrain from conducting all pre-specified moderator analyses. Further limitations are based on characteristics of the included studies themselves. A major problem was the small sample size of the selected studies; only four studies included more than 50 subjects leading to imprecision of the results.

Another critical issue is that the internal validity of the included studies was prone to bias, since the overall risk-of-bias judgment was ‘some concerns’ in all studies and ‘high risk’ of bias within single domains was rated for three studies. This was particularly true for biases due to deviations from the intended intervention, the measurement of the outcome, and selective reporting. However, excluding studies with high risk of bias in any domain [46, 47, 51] did not change the results or our conclusions.

## Conclusions

This is the first meta-analysis of the efficacy of AT on the reduction of chronic pain. The results of this meta-analysis indicate that AT can be used as an effective relaxation technique for individuals suffering from chronic pain. AT is comparably efficacious in reducing pain as other psychological interventions, e.g., progressive muscle relaxation or self-hypnosis. However, our confidence in this conclusion is limited because of restricted internal validity of the included studies. Moreover, questions about differential efficacy of AT remain unanswered. Based on the low number of included studies and the respective low statistical power, not all pre-specified subgroup and meta-regression analyses were conducted. Hence, future studies should apply more rigorous research methods that ensure high internal validity to strengthen the promising findings of the efficacy of AT in pain reduction and to additionally clarify, for whom and in which circumstances AT works best.

## Supplementary Information

Below is the link to the electronic supplementary material.

Supplementary file1 (DOCX 142 KB)
