# Supplementary material for: Autogenic Training for Reducing Chronic Pain: a Systematic Review and Meta-analysis of Randomized Controlled Trials
Source: Int J Behav Med. 2021 Oct 27;29(5):531–42. doi: 10.1007/s12529-021-10038-6 (PMC8548271; doi:10.1007/s12529-021-10038-6)
Supplement: Supplementary file 1 — Supplementary file1 (DOCX 142 KB) [file 12529_2021_10038_MOESM1_ESM.docx]

**Supplementary Material**

*Search Terms for Medline (Date of Last Search: 04/07/2021)*

#19 “(#3 AND #13 AND #18)”

#18 “(#14 OR #15 OR #16 OR #17)”

#17 “(randomly[Title/Abstract] OR trial[Title/Abstract])”

#16 “clinical trial as topic[MeSH Terms]”

#15 “(randomized OR controlled OR placebo)”

#14 “(controlled clinical trial OR randomized controlled trial[MeSH Terms])”

#13 “(#4 OR #5 OR #6 OR #7 OR #8 OR #9 OR #10 OR #11 OR #12)”

#12 “(somatoform disorder* OR fibromyalg* OR chronic fatigu* OR arthrit* OR

headache* OR migrain* OR labor* OR pregnan* cancer OR temporomandibul* OR

sickle cell OR multiple sclero* OR asthma* OR raynaud* OR inflammatory bowel* OR

epilep* OR eczem* OR HIV* OR diabet* OR tinnitus* OR dysmenorrhea* OR irritable

bowel* OR stroke* OR ulcera* colit* OR burn* OR wound* OR injur* OR sciatic* OR

disc displacement* OR inflammation* OR herpes zoster OR shingle* OR spinal Injur*

OR spinal cord injur* OR amputation* OR alcoholism* OR alcohol abuse)”

#11 “(sciatic n europathy OR inflammation OR herpes zoster OR amputation OR Substance-Related Disorders[MeSH Terms])”

#10 “(diabetes mellitus OR tinnitus OR irritable bowel syndrome OR stroke OR wounds and injuries[MeSH Terms])”

#9 “(cardiovascular diseases OR digestive system diseases OR epilepsy OR skin diseases OR HIV[MeSH Terms])”

#8 “(hemic and lymphatic diseases OR palliative care OR multiple sclerosis OR asthma[MeSH Terms])”

#7 “(musculoskeletal diseases OR arthritis OR headache disorder OR labor OR pregnancy OR neoplasms[MeSH Terms])”

#6 “(somatoform disorder OR fibromyalgia OR chronic fatigue syndrom[MeSH Terms])”

#5 “pain*”

#4 “pain[MeSH Terms]”

#3 “(#1 OR #2)”

#2 “(autogenic* OR autosuggest*)”

#1 “(autogenic training OR autosuggestion[MeSH Terms])”

*Search Terms for EBSCOhost (Date of Last Search: 04/07/2021)*

#22 #19 AND #20 AND #21

#21 #1 OR #2 OR #3 OR #4

#20 #5 OR #6 OR #7 OR #8

#19 #9 OR #10 OR #11 OR #12 OR #13 OR #14 OR #15 OR #16 OR #17 OR #18

#18 MR "treatment outcome"

#17 MR "clinical trial"

#16 MR "meta analysis"

#15 MR "empirical study"

#14 TI control* OR AB control* OR KW control*

#13 TI random* OR AB random* OR KW random*

#12 DE "Clinical Trials"

#11 DE "Between Groups Design"

#10 DE "experiment controls"

#9 DE "random sampling"

#8 DE ( dysmenorrhea* OR irritable bowel* OR stroke OR ulcerat* colit* OR burn* OR wound* OR injur* OR operat* OR surg* OR vascular* OR sciatic* OR disc displace* OR inflammation* OR herpes zoster OR shingles* OR (spinal N5 injur*) OR amputation* OR alcoholism* OR alcohol abuse* ) OR TI ( dysmenorrhea* OR irritable bowel* OR stroke OR ulcerat* colit* OR burn* OR wound* OR injur* OR operat* OR surg* OR vascular* OR sciatic* OR disc displace* OR inflammation* OR herpes zoster OR shingles* OR (spinal N5 injur*) OR amputation* OR alcoholism* OR alcohol abuse* ) OR AB ( dysmenorrhea* OR irritable bowel* OR stroke OR ulcerat* colit* OR burn* OR wound* OR injur* OR operat* OR surg* OR vascular* OR sciatic* OR disc displace* OR inflammation* OR herpes zoster OR shingles* OR (spinal N5 injur*) OR amputation* OR alcoholism* OR alcohol abuse* ) OR KW ( dysmenorrhea* OR irritable bowel* OR stroke OR ulcerat* colit* OR burn* OR wound* OR injur* OR operat* OR surg* OR vascular* OR sciatic* OR disc displace* OR inflammation* OR herpes zoster OR shingles* OR (spinal N5 injur*) OR amputation* OR alcoholism* OR alcohol abuse* )

#7 DE ( multiple sclero* OR asthma* OR raynaud* OR inflammatory bowel* OR epilep* OR eczem* OR HIV OR cardiovascular* OR diabetes* OR tinnitus* ) OR TI ( multiple sclero* OR asthma* OR raynaud* OR inflammatory bowel* OR epilep* OR eczem* OR HIV OR cardiovascular* OR diabetes* OR tinnitus* ) OR AB ( multiple sclero* OR asthma* OR raynaud* OR inflammatory bowel* OR epilep* OR eczem* OR HIV OR cardiovascular* OR diabetes* OR tinnitus* ) OR KW ( multiple sclero* OR asthma* OR raynaud* OR inflammatory bowel* OR epilep* OR eczem* OR HIV OR cardiovascular* OR diabetes* OR tinnitus* )

#6 DE ( somatoform* OR fibromyalg* OR analge* OR chronic fatig* OR *arthrit* OR rheuma* OR back ache* OR head ache* OR migrain* OR parturition* OR childbirth* OR neoplasm* OR joint disorder* OR sickle cell* OR palliative care* ) OR TI ( somatoform* OR fibromyalg* OR analge* OR chronic fatig* OR *arthrit* OR rheuma* OR back ache* OR head ache* OR migrain* OR parturition* OR childbirth* OR neoplasm* OR joint disorder* OR sickle cell* OR palliative care* ) OR AB ( somatoform* OR fibromyalg* OR analge* OR chronic fatig* OR *arthrit* OR rheuma* OR back ache* OR head ache* OR migrain* OR parturition* OR childbirth* OR neoplasm* OR joint disorder* OR sickle cell* OR palliative care* ) OR KW ( somatoform* OR fibromyalg* OR analge* OR chronic fatig* OR *arthrit* OR rheuma* OR back ache* OR head ache* OR migrain* OR parturition* OR childbirth* OR neoplasm* OR joint disorder* OR sickle cell* OR palliative care* )

#5 DE pain OR TI pain OR AB pain OR KW pain

#4 DE ( autogenic NOT drainag* ) OR TI ( autogenic NOT drainag* ) OR AB ( autogenic NOT drainag* ) OR KW ( autogenic NOT drainag* )

#3 DE autosuggestion OR TI autosuggest* OR AB autosuggest* OR KW autosuggest*

#2 (TI ( autogenic training* OR autogenic relaxation* OR autogenic suggest* OR autogenic therap* OR autogenic treat* OR autogenic meditat* OR autogenic feedback*) OR AB (autogenic training* OR autogenic relaxation* OR autogenic suggest* OR autogenic therap* OR autogenic treat* OR autogenic meditat* OR autogenic feedback* ) OR KW ( autogenic training* OR autogenic relaxation* OR autogenic suggest* OR autogenic therap* OR autogenic treat* OR autogenic meditat* OR autogenic feedback*) )

#1 DE autogenic training

*Search Terms for CENTRAL (Date of Last Search: 04/07/2021)*

#1 MeSH descriptor: [Autogenic Training] explode all trees

#2 MeSH descriptor: [Autosuggestion] explode all trees

#3 autogenic relaxation

#4 autogenic therapy

#5 autogenic meditation

#6 autogenic suggestion

#7 autogenic feedback

#8 autogenic

#9 #1 OR #2 OR #3 OR #4 OR #5 OR #6 OR #7 OR #8

#10 MeSH descriptor: [Pain] explode all trees

#11 MeSH descriptor: [Acute Pain] explode all trees

#12 MeSH descriptor: [Chronic Pain] explode all trees

#13 MeSH descriptor: [Somatoform Disorders] explode all trees

#14 analge*

#15 MeSH descriptor: [Fibromyalgia] explode all trees

#16 MeSH descriptor: [Arthritis, Rheumatoid] explode all trees

#17 MeSH descriptor: [Osteoarthritis] explode all trees

#18 MeSH descriptor: [Back Pain] explode all trees

#19 MeSH descriptor: [Migraine Disorders] explode all trees

#20 MeSH descriptor: [Tension-Type Headache] explode all trees

#21 MeSH descriptor: [Parturition] explode all trees

#22 MeSH descriptor: [Labor Pain] explode all trees

#23 MeSH descriptor: [Neoplasms] explode all trees

#24 MeSH descriptor: [Temporomandibular Joint Disorders] explode all trees

#25 MeSH descriptor: [Anemia, Sickle Cell] explode all trees

#26 MeSH descriptor: [Palliative Care] explode all trees

#27 MeSH descriptor: [Multiple Sclerosis] explode all trees

#28 operati*

#29 surg*

#30 MeSH descriptor: [Asthma] explode all trees

#31 MeSH descriptor: [Raynaud Disease] explode all trees

#32 MeSH descriptor: [Inflammatory Bowel Diseases] explode all trees

#33 MeSH descriptor: [Epilepsy] explode all trees

#34 MeSH descriptor: [Eczema] explode all trees

#35 MeSH descriptor: [HIV] explode all trees

#36 MeSH descriptor: [Cardiovascular Diseases] explode all trees

#37 MeSH descriptor: [Diabetes Mellitus] explode all trees

#38 MeSH descriptor: [Tinnitus] explode all trees

#39 MeSH descriptor: [Dysmenorrhea] explode all trees

#40 MeSH descriptor: [Irritable Bowel Syndrome] explode all trees

#41 MeSH descriptor: [Stroke] explode all trees

#42 MeSH descriptor: [Colitis, Ulcerative] explode all trees

#43 MeSH descriptor: [Burns] explode all trees

#44 MeSH descriptor: [Vascular Diseases] explode all trees

#45 MeSH descriptor: [Wounds and Injuries] explode all trees

#46 MeSH descriptor: [Sciatic Neuropathy] explode all trees

#47 MeSH descriptor: [Intervertebral Disc Displacement] explode all trees

#48 MeSH descriptor: [Inflammation] explode all trees

#49 MeSH descriptor: [Herpes Zoster] explode all trees

#50 MeSH descriptor: [Spinal Injuries] explode all trees

#51 MeSH descriptor: [Spinal Cord Injuries] explode all trees

#52 MeSH descriptor: [Amputation] explode all trees

#53 MeSH descriptor: [Alcoholism] explode all trees

#54 #10 OR #11 OR #12 OR #13 OR #14 OR #15 OR #16 OR #17 OR #18 OR #19 OR #20

OR #21 OR #22 OR #23 OR #24 OR #25 OR #26 OR #27 OR #28 OR #29 OR #30 OR

#31 OR #32 OR #33 OR #34 OR #35 OR #36 OR #37 OR #38 OR #39 OR #40 OR #41

OR #42 OR #43 OR #44 OR #45 OR #46 OR #47 OR #48 OR #49 OR #50 OR #51 OR

#52 OR #53

#55 #9 AND #54

*Search Terms for Web of Science (Date of Last Search: 04/07/2021)*

#11 #10 NOT TS=(autogenic drainage*)

#10 #9 NOT TS=(animals OR rats OR mice)

#9 #8 AND #7 AND #3

#8 TS=(random* OR control*)

#7 #6 OR #5 OR #4

#6 TS=(somatoform disorder* OR fibromyalg* OR chronic fatig* OR analge* OR rheuma* OR arthrit* OR back ache* OR migraine* OR headache* OR parturition* OR labor* OR neoplasm* OR joint disorder* OR sickle cell* OR palliative care* OR multiple sclera* OR operatio* PR surg* OR asthma* OR raynaud* OR inflammatory bowel* OR epilep* OR eczem* OR HIV* OR cardiovascular* OR diabetes* OR tinnitus* OR dysmenorrhea* OR irritable bowel* OR stroke* OR ulcerat* colit* OR burns OR vascular* OR wound* OR injur* OR sciatic* OR disc displacement* OR inflammation* OR herpes zoster OR shingles OR spinal cord injur* OR spinal injur* OR amputation* OR alcoholism* OR alcohol abuse*)

#5 TS=((acute* OR chronic* OR somatoform*) near/5 (pain*))

#4 TS=(pain*)

#3 #2 OR #1

#2 TS=(autogenic* OR autosuggest*)

#1 TS=((autogenic*) near/5 (training* OR therap* OR relaxation* OR treatment* OR meditation* OR suggest* OR feedback*))

Supplementary Table 1

*Risk of Bias Judgements for Each Included Study*

|  |  | Randomization process | Deviations from intended interventions | Missing outcome data | Measurement of the outcome | Selection of the reported result | Overall risk |
| --- | --- | --- | --- | --- | --- | --- | --- |
| Passive control groups | Asbury et al. (2009) |  |  |  |  |  |  |
|  | Engel et al. (1990) |  |  |  |  |  |  |
|  | Labbé (1995) |  |  |  |  |  |  |
|  | Mantovani et al. (1996) |  |  |  |  |  |  |
|  | Pickering et al. (2012) |  |  |  |  |  |  |
|  | Sargent et al. (1986) |  |  |  |  |  |  |
|  | Shinozaki et al. (2009) |  |  |  |  |  |  |
|  | Sutherland et al. (2005) |  |  |  |  |  |  |
|  | ter Kuile et al. (1994) |  |  |  |  |  |  |
|  |  |  |  |  |  |  |  |
| Active control groups | Bernateck et al. (2008) |  |  |  |  |  |  |
|  | Collet et al. (1986) |  |  |  |  |  |  |
|  | Engel et al. (1990) |  |  |  |  |  |  |
|  | Janssen & Neutgens (1986) |  |  |  |  |  |  |
|  | Spinhoven et al. (1992) |  |  |  |  |  |  |
|  | ter Kuile et al. (1994) |  |  |  |  |  |  |

*Note*. Low risk of bias*;* Some concerns; High risk of bias

Supplementary Table 2

*Moderator Analyses for the Primary Outcome Pain*

|  | *k* | *g* | 95% *CI* | *p (g)* | *Q* | *p (Q)* | *I^2^* | *p_diff_* |
| --- | --- | --- | --- | --- | --- | --- | --- | --- |
| Intervention format |  |  |  |  |  |  |  | 0.677 |
| Individual AT | 7 | 0.33 | 0.01; 0.65 | 0.041 | 3.44 | 0.752 | 0% |  |
| AT in groups | 7 | 0.24 | -0.08; 0.55 | 0.137 | 16.74 | 0.010 | 64% |  |
| Intervention mode |  |  |  |  |  |  |  | 0.161 |
| Live | 6 | 0.49 | 0.16; 0.82 | 0.004 | 8.53 | 0.130 | 41% |  |
| Audio or Live + Audio | 9 | 0.19 | -0.08; 0.45 | 0.165 | 10.28 | 0.246 | 22% |  |
|  | *k* | *β* | *SE* | *p* | *R^2^* |  |  |  |
| Intervention duration (min) | 14 | 0.0005 | 0.0003 | 0.093 | 0.34 |  |  |  |

Supplementary Table 3

*Results of Sensitivity Analyses for the Primary Outcome Pain*

|  | *k* | *g* | 95% *CI* | *p (g)* | *Q* | *p (Q)* | *I^2^* |
| --- | --- | --- | --- | --- | --- | --- | --- |
| Overall effect | 15 | 0.30 | 0.09; 0.51 | 0.005 | 21.35 | 0.093 | 34% |
| Approximated ES excluded | 11 | 0.33 | 0.09; 0.58 | 0.008 | 16.81 | 0.079 | 41% |
| Excluding studies with children | 12 | 0.29 | 0.06; 0.52 | 0.015 | 20.05 | 0.045 | 45% |
| Excluding high risk of bias studies | 12 | 0.32 | 0.11; 0.54 | 0.003 | 14.92 | 0.186 | 26% |
| Passive control groups | 9 | 0.58 | 0.36; 0.79 | <0.001 | 3.28 | 0.916 | 0% |
| Approximated ES excluded | 7 | 0.59 | 0.35; 0.82 | <0.001 | 3.22 | 0.781 | 0% |
| Excluding studies with children | 7 | 0.57 | 0.35; 0.79 | <0.001 | 2.90 | 0.821 | 0% |
| Excluding high risk of bias studies | 7 | 0.57 | 0.33; 0.81 | <0.001 | 2.95 | 0.815 | 0% |
| Active control groups | 6 | -0.05 | -0.30; 0.20 | 0.692 | 4.15 | 0.527 | 0% |
| Approximated ES excluded | 4 | -0.01 | -0.29; 0.27 | 0.947 | 3.60 | 0.309 | 17% |
| Excluding studies with children | 5 | -0.05 | -0.31; 0.20 | 0.684 | 4.15 | 0.387 | 4% |
| Excluding high risk of bias studies | 5 | 0.01 | -0.26; 0.29 | 0.925 | 2.79 | 0.593 | 0% |

ES = effect sizes

Supplementary Figure 1

*Funnel Plot of Standard Error by Fisher´s Z*

-2.0

-1.5

-1.0

-0.5

0.0

0.5

1.0

1.5

2.0

0.0

0.2

0.4

0.6

0.8

Standard Error

Hedges' g

*Note:* No studies were “missing”. Hence, the unfilled diamond representing the observed effect corresponds to the filled diamond showing the adjusted effect.
